# Low dose DNA methyltransferase inhibitors potentiate PARP inhibitors in homologous recombination repair deficient tumors

**DOI:** 10.1186/s13058-024-01954-y

**Published:** 2025-01-16

**Authors:** Romain Pacaud, Scott Thomas, Sibapriya Chaudhuri, Ann Lazar, Luika A. Timmerman, Pamela N. Munster

**Affiliations:** 1 Department of Medicine (Hematology/Oncology), School of Medicine, University of California San Francisco, 1450 Third St, San Francisco, CA 94158 USA; 2 Division of Oral Epidemiology and Division of Biostatistics, School of Dentistry and School of Medicine, University of California San Francisco, San Francisco, CA USA; 3 Helen Diller Family Comprehensive Cancer Center, University of California San Francisco, San Francisco, CA USA

**Keywords:** BRCA1, BRCA2, PARP inhibitor, DNMT inhibitor, Talazoparib, Decitabine, PARP1, DNMT1

## Abstract

**Background:**

Poly (ADP-Ribose) polymerase inhibitors are approved for treatment of tumors with BRCA1/2 and other homologous recombination repair (HRR) mutations. However, clinical responses are often not durable and treatment may be detrimental in advanced cancer due to excessive toxicities. Thus we are seeking alternative therapeutics to enhance PARP-directed outcomes. In an effort to expand the clinical use of PARP inhibitors to HRR proficient tumors, several groups have tested combinations of DNA methyltransferase inhibitors and PARP inhibitors. While this approach attenuated tumor cell proliferation in preclinical studies, subsequent clinical trials revealed little benefit. We hypothesized that benefit for this drug combination would only be specific to HRR deficient tumors, due to their inability to enact high fidelity DNA repair with subsequent cell death.

**Methods:**

We generated hypomorphic BRCA1 and BRCA2 variants of the HRR proficient triple negative breast cancer cell line MDA-MB-231. We compared therapeutic response features such as RAD51 focus formation, cell cycle fraction alterations, DNA damage accumulation, colony formation, and cell death of these and other cell lines with and without intrinsic BRCA1/2 mutations. Results were confirmed in BRCA1/2 intact and deficient xenografts and PDX.

**Results:**

Our targeted variants and cells with intrinsic BRCA1/2 mutations responded to low dose combination therapeutic treatment by G2M stalling, compounded DNA damage, severely attenuated colony formation, and importantly, increased cell death. In contrast, the parental MDA-MB-231 cells and other HRR proficient cell lines produced smaller cell populations with short term treatment, but with much less cumulative DNA damage, and minimal cell death. In animal studies, our BRCA-engineered hypomorphs and several independent PDX models with clinically relevant BRCA mutations were acutely more vulnerable to this drug combination.

**Conclusions:**

We conclude that low dose DNA methyltransferase inhibition can cooperate with low dose PARP inhibition to increase DNA damage predominantly in cells with HRR deficiencies, ultimately producing more cell death than in HRR proficient tumors. We predict that clinical benefit will more likely be apparent in patients with DNA repair defective tumors and are focusing clinical exploration of this drug combination in these patients, with the goals of enhancing tumor cell death at minimal toxicities.

**Supplementary Information:**

The online version contains supplementary material available at 10.1186/s13058-024-01954-y.

## Background

In the recent CARRIERS study, the prevalence of BRCA1 and BRCA2 mutations in women with breast cancer were 2.14% versus 0.35% in non-breast cancer controls [1]. BRCA mutations in women lead to a 40–80% lifetime risk of breast cancer with about 40% of women diagnosed under the age of 40 [2, 3]. *BRCA1* and *BRCA2* are the most common germline variants in breast, ovary, pancreas, and prostate cancer [4]. Pathogenic variants in *BRCA1* and *BRCA2* are suggested to impair DNA double strand break (DSB) repair by homologous recombination repair (HRR; review: [5]). BRCA1 and BRCA2-containing complexes recruit and stabilize RAD51 proteins on trimmed, single strand DNA probes at DNA damage sites. This facilitates base pairing with homologous sequences on a sister chromatid to ultimately produce high fidelity repair. The ability to form RAD51 foci correlates strongly with the ability to repair DSBs by HRR [6], and early clinical studies use the inability to form RAD51 foci as a measure of functional HRR deficiency [7]. Deficient repair has been successfully exploited therapeutically by inhibition of Poly (ADP-Ribose) Polymerase 1 and 2 (PARP1 and PARP2; review [8, 9]).

PARP-containing complexes repair DNA single strand breaks (SSBs) and mend un-ligated Okazaki fragments during normal DNA synthesis (reviews: [10, 11]). PARP1 further protects replication forks from degradation when DNA polymerase progression stalls, and protects unresolved SSBs from conversion to toxic DSBs. Clinically, one of the most potent PARP inhibitors (PARPi) is talazoparib, which additionally traps PARP1 onto DNA at SSBs by preventing auto-PARylation and DNA release (reviews: [12, 13]). Although many of the subsequent details of its mechanism of action remain unclear, currently accepted models propose that trapped PARPi-PARP1complexes erect a polymerase barrier at replication forks, inducing DSBs and low fidelity DNA repair in HRR deficient cells [13, 14]. While highly effective in HRR mutated breast, ovarian, prostate, and pancreatic cancer, the benefits often remain short-lived. Combinations with other cytotoxic agents have proven to be too toxic due to the narrow therapeutic window of PARPi. Our studies are directed towards finding combination strategies that could enhance PARPi efficacy by mechanistically exploiting the mode of actions of PARP inhibitors such as talazoparib and a second drug that could enhance efficacy at low concentrations, specifically for patients with HRR deficient tumors.

DNA methyltransferases (DNMTs) modify DNA by adding methyl groups on the fifth carbon atom of cytosine nucleotides immediately 5′ of guanines (CpG). CpG dinucleotides are enriched in gene promoters, where methylation can silence genes such as tumor suppressors [15]. Methylation of repetitive genomic elements also prevents chromosomal instability [16]. DNMT1 has roles in DNA repair [17], and maintenance of DNA methylation patterns during replication (review: [18]). The DNMT inhibitory (DNMTi) cytosine analogs 5-azacitidine (5-AZA) and 5-aza-2′-deoxycytidine (decitabine) are approved in myelodysplastic syndrome (reviews: [19, 20]). Azacytidine is primarily incorporated into RNA, with about 20% converted to the cytosine analog 5-aza-2′-deoxycytidine for DNA incorporation in place of cytosine [21]. Decitabine is only incorporated into DNA. Prior preclinical studies suggest that both PARPi and DNMTi generate stable complexes at DSBs and replication forks, yielding increased DNA damage [22]. This drug combination also significantly reduced growth of HRR proficient cell lines in vitro and in xenograft, potentially greatly expanding the clinical use of PARP inhibitors [22–24]. Unfortunately, exploration of DNMTi and PARPi combinations in two clinical studies in patients with intact HRR status demonstrate limited actual benefit ([25], and ClinicalTrials.gov NCT04134884 personal communication).

We speculated that patients with HRR deficient tumors might be the true beneficiaries of such a drug combination, reasoning that while HRR proficient tumor cells can perform error free DNA repair to cleanly traverse the cell cycle, HRR deficient tumors can only enact error-prone repair. Over time and through multiple cell cycles, these latter tumors should therefore sustain increasing amounts of DNA damage, ultimately more likely enhancing cell death.

## Methods

### Pharmaceuticals

Talazoparib (Cat. #S7048), decitabine (Cat. #S1200), and carboplatin (Cat. #S1215) were from SelleckChem®. 10 mM stocks were made in DMSO.

### Cell culture

MDA-MB-231 (M231^wt^), and MCF10A were from ATCC; SUM149PT from Applied Biosciences; BT20 from Dr. Joe Gray, University of Oregon Health Sciences Center; JHOS2 and COV362 from Dr. Wendy Fantl, Stanford University. M231 media: RPMI-1640/10% fetal bovine serum (FBS)/1X penicillin /streptomycin (P/S)/ 2 mM L-glutamine. MCF10A media: DMEM/F12/ 5% horse serum/ 20 ng/ml EGF/ 0.5 mg/ml hydrocortisone/ 100 ng/ml cholera toxin/ 10 µg/ml insulin/ 1X P/S. SUM149PT media: Ham’s F-12/ 5% FBS/ 1X P/S/ 1 µg/mL hydrocortisone/ 5 µg/mL insulin. BT20 media: RPMI-1640/ 10% FBS. JHOS2 media: DMEM/F12/ 10% FBS/ 1% MEM NEAA/ 1X P/S. COV362 media: DMEM/ 10% FBS/ 1X P/S. All cells were maintained in a humidified incubator at 37˚C with 5% CO_2_. For all assays, cells were seeded and allowed to adhere overnight (37˚C, 5% CO_2_) before use. Unless indicated otherwise, cells were treated with talazoparib (0–5 nM), decitabine (0–14 nM), the combination, or DMSO.

### Proliferation

1000 cells/well in 96-well plates were treated for 6 days (n = 4). Final cell number determined with the sulphorhodamine B endpoint assay kit (SRB; G-Biosciences Cat. #786-213). Synergy determined by the SynergyFinder web tool [26].

### ***IC***_***50***_*** determination***

1 × 10^4^ SUM149PT cells/well were treated for 48 h with indicated drug doses, and cell numbers derived using CellTiter 96® Aqueous Non-Radioactive Cell Proliferation Assay (Promega®). The IC_50_ was determined by nonlinear regression analysis using Prism® (GraphPad).

### Immunofluorescence

1 × 10^4^ cells/well were seeded onto coverslips and treated. At indicated times coverslips were fixed (4% paraformaldehyde), permeabilized (PBS/0.2% Triton X-100), blocked (PBS/2% BSA/1 h), and stained with 1:100 fluorescently labeled anti-phosphorylated-histone H2A.X (p-H2A.X; Ser139; #CR55T33, eBioscience®). Coverslips were mounted with Duolink In Situ Mounting Medium with 4′,6-diamidino-2-phenylindole (DAPI; Sigma-Aldrich®). Imaging and analysis used a Zeiss spinning disk Observer Z1 confocal microscope (Zeiss®), coupled with a CSU-X1 Confocal Scanner (YOKOGAWA®), with Z-stack images acquisition. Z-stack files were analyzed (CellProfiler, [27]) using an approach that segregates nuclei by DAPI staining through Z-stack reconstructions and counts the number of fluorescent spots per complete nucleus (50 nuclei total per observation/condition, n = 50). Foci numbers per nucleus were analyzed using Prism® (GraphPad).

### Enumeration of micronuclei

Cells were plated on coverslips and allowed to adhere overnight. Subsequently, 5 nM talazoparib + 15 nM decitabine, or DMSO were applied, and coverslips harvested daily and formalin fixed (2%) over 5 days. Coverslips were PBS rinsed, blocked (PBS/2% BSA/1 h), fixed (3% formalin), and stained for CD44 (BD Pharmingen 561,860) to outline cell membranes, and DAPI to stain nuclear material. Images were acquired at 40X and 63X, and cells and nuclear abnormalities tabulated by hand, three times, for at least 500 cells per condition. Results from day 4 treatment are presented.

### *Proximity-ligation *in situ* assay for RAD51 foci*

Following the indicated treatment, cells were blocked, stained, hybridized, ligated, amplified, and detection performed according to manufacturer's instructions (#DUO92208, Sigma-Aldrich®). Anti-RAD51 (#ABE257, Millipore and #sc-53428, Santa Cruz Biotechnology) were used.

### Cell death assay

2 × 10^4^ cells/well in 6-well plates were treated for 6 days. Adherent and non-adherent cells were harvested and analyzed for cell death using the Calcein AM Live and Dead Cell Assay (#ab115347, Abcam) and flow cytometry (Attune NxT, Thermo Scientific). Data analysis used FlowJo (BD Biosciences).

### Cell cycle analysis

1 × 10^5^ cells/well in 6-well plates were treated for 48 h, fixed with 66% ethanol/4C/2 h, and stored at 4C. Nuclei were stained using the propidium iodide flow cytometry kit (#ab139418, Abcam) per the manufacturers’ protocol. Briefly, fixed cells were stained with 200µL 1X propidium iodide + RNase A, incubated at 37˚C for 30 min in the dark, and analyzed on a flow cytometer (FACSVerse, BD Biosciences). Data analysis used FlowJo (BD Biosciences).

### Clonogenic assay

500 cells/well in 6-well plates were treated with media and drug exchange every 48 h for 10 days. Colonies were fixed/stained 30 min with 0.5% crystal violet/20% methanol and images qualitatively acquired with a BioRad ChemiDoc imaging System (BioRad®), with color correction and exposure time alteration to allow optimum colony identification using Image Lab software (BioRad®) and quantitatively assessed by ImageJ (FiJi). These values were normalized to control treated wells and graphed with Prism® (GraphPad).

### CRISPR/Cas9 and sequencing

The HRR proficient cell line M231 was genetically modified using a CRISPR/Cas9 system [28]. Briefly, oligonucleotides targeting *BRCA1* and *BRCA2* were ligated into the plasmid pSpCas9(BB)-2A-Puro (PX459; Addgene plasmid #62,988) at the BbsI site (BbsI-HF; cat. #R3539, New-England Biolabs®) and transformed into DH5α, (Cat. #18,265,017, Invitrogen®). The forward primer included the anchor 5′-CACC(G)-N_(20)_-3′, and the reverse primer included the 5′-AAAC-N_(20)_-C-3′ anchor. Plasmids were purified (Zyppy Plasmid MiniPrep, Cat. #D4019, Zymo Research®) and transfected into M231 cells (Lipofectamine LTX with PLUS reagent, Cat. #A12621, Invitrogen®), according to manufacturer protocol. Cells incorporating the plasmid were selected by culture with 1 µg/mL puromycin dihydrochloride (Cat. #A1113803, Gibco ®) for 48–72 h. Surviving cells were subcloned into a 96-well plate by FACS using the single cell sorting mode (SH800, Sony®), gating on FSC-A/SSC-A, then FSC-A/FSC-W to identify healthy singlet cells. Clonal cultures were cultured for expansion and tested by DNA extraction, PCR, and Sanger sequencing to identify clones with targeted deletions in all alleles. Allele assembly and analysis used the web tool DSDecode [29].

Primer sequences are shown in the following chart.

**Table:** 

Guide Oligo name	Sequence (5′—>3′)
BRCA1 sgRNA Forward	CACCGAACTCTGAGGACAAAGCAG
BRCA1 sgRNA Reverse	AAACCTGCTTTGTCCTCAGAGTTC
BRCA2 sgRNA Forward	CACCGCTGTACCAATCTCCTGTAAA
BRCA2 sgRNA Reverse	AAACTTTACAGGAGATTGGTACAGC
BRCA1 CRISPR target zone Forward	CACTCTGTTGCTTATGCTGG
BRCA1 CRISPR target zone Reverse	TTCACTTCCCAAAGCTGCCTAC
BRCA2 CRISPR target zone Forward	AGCTCCACCCTATAATTCTGAACC
BRCA2 CRISPR target zone Reverse	CAGAGAGACTGATTTGCCCAGC

### Xenograft and PDX

Six to seven week old female NOD.Cg-Rag1^tm1Mom^ Il2rg^tm1Wjl^/SzJ mice (Strain #007799, The Jackson Laboratory) were used to avoid sensitivity to DNA damaging therapies associated with other immune compromised strains. 3 × 10^6^ tumor cells were injected in Matrigel (Cat. #CLS354230, Corning®), into mammary fatpads or 2–5 mm^3^ PDX tumor fragments subcutaneously implanted from BRCA1 mutant breast cancer PDX models TM00089 and TM00091 (Jackson Research Labs). When tumors reached ~ 100 mm^3^, mice were randomly assigned to 4 treatment groups. Xenografts recieved 0.1 mg/kg talazoparib (Cat. #S7048, SelleckChem®) given by oral gavage ± 0.2 mg/kg decitabine (Cat. #S1200, SelleckChem®) given by intraperitoneal (IP) injection. PDX received the identical talazoparib dose, and ½ dose of decitabine. Treatment schedule for all animals: 5-days on/2-days off. Animal weights and tumor volumes were assessed twice per week. Tumor volumes calculated as (length x width^2)^/2). Stock talazoparib in 10 mM DMSO was diluted in 10% dimethylacetamide (Cat. #271,012, Sigma-Aldrich®), 6% Kolliphor HS 15 (Cat. #42,966, Sigma-Aldrich®), and 84% PBS. Stock decitabine in 10 mM DMSO was diluted in PBS. Vehicle only animals received an oral gavage mixture without talazoparib, and IP PBS/DMSO. All experiments were conducted under institutional animal care and use committee approval (AN180895-03C). Experiments were stopped when control animal tumor volumes became > 1500 mm^3^ per institutional guidelines.

### Statistical analyses

All statistical methods are described within the figure legends. Mean ± SD were calculated for the SRB endpoint assays, cell cycle assays, and cell death assays. Treatment group comparisons for SRB assays and the SUM149PT cell death assay were generated via 1-way ANOVA, significant differences for the M231 variants death assay was calculated by 2-way ANOVA. The means ± SEM were calculated for p-H2A.X foci, colony counts, xenograft, and PDX experiments. Significant differences between time and treatment groups for p-H2A.X foci counts were calculated by 2-way ANOVA. Significant differences between colony numbers were calculated by 1-way ANOVA. Significant differences between xenografts and PDX treatment groups were calculated by 2-way ANOVA. All analyses used Tukey’s adjustment for multiple testing. All experiments except the animal studies were performed at least twice. Each data point represents at least triplicate measurements, with a single representative experiment shown. Statistics and graphs used Prism® (GraphPad).

## Results

### Generation and characterization of isogenic BRCA mutant cell lines

We hypothesized that HRR deficiency would be a key regulator of cell death in response to talazoparib (Tala) + decitabine (Deci) treatment. To test this we selectively targeted the *BRCA1* and *BRCA2* loci in the HRR proficient triple negative breast cancer cell line MDA-MB-231 (M231^*wt*^), using standard CRISPR-Cas9 techniques [28] to generate an isogenic cell line panel. Single guide RNAs targeting the 5′end of *BRCA1* between the nuclear export sequences (amino acids (aa) 81–99) and the nuclear import sequences (aa 200–300) generated modest biallelic mutations in clone B1.85 (M231^*BRCA1*^; Supplementary Fig. S1A; [29]). A 42 base pair deletion in allele 1 produced a leucine > histidine substitution at aa 165, and deleted 14 subsequent amino acids (to aa 180). This potentially compromises the MB2 myc binding site (aa 175–300; [30]). A simple 3 base pair deletion at the CRISPR guide target site in allele 2 caused deletion of glutamine at aa 169. We found BRCA1 glutamine 169 variants on ClinVar [31] but no deletion mutations at aa 169, nor leucine to histidine conversion at 165, nor larger deletions containing the missing14 amino acids in our clone.

Our BRCA2 guides targeted sequence within the transactivation domain (aa 15-105; [32]), just 3′ of the PALB2 binding site**.** Clone B2A (M231^*BRCA2*^) also had biallelic *BRCA2* mutations: the first, a 22-base pair deletion that induced a frameshift stop after aa 90 (Supplementary Fig. S1B). The second allele had a 33 base pair deletion that removed aa 86–96 from the transactivation domain. Thus, it is possible that at least transactivation domain function in this clone may be compromised. These deletion mutations are not found on ClinVar.

**Fig. 1:**
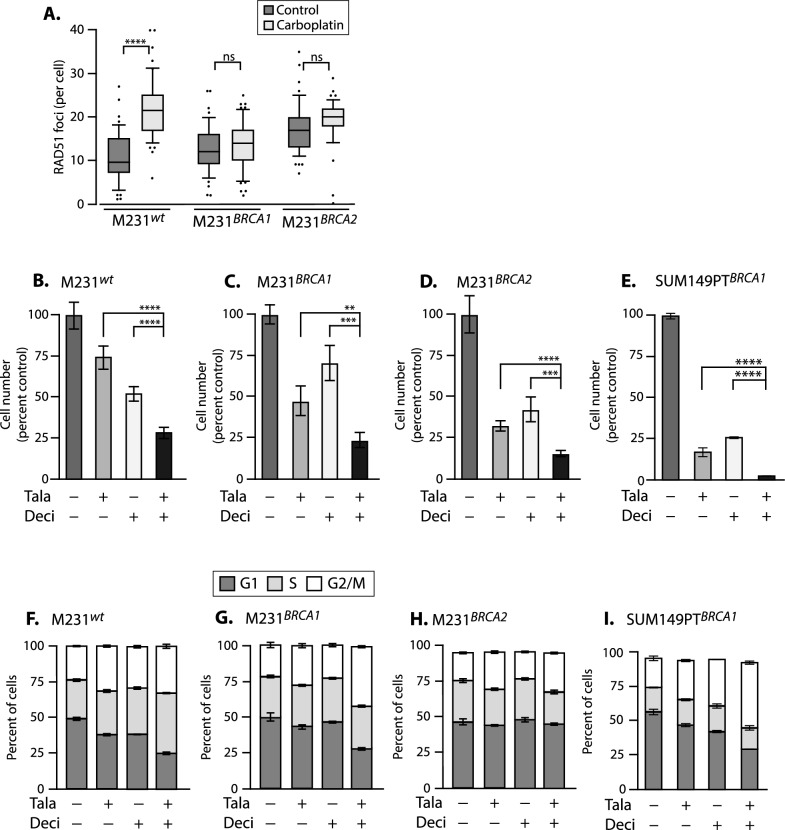
Talazoparib and decitabine synergize in treatment of M231^*wt*^ and isogenic *BRCA1* and *BRCA2*-targeted mutants. **A** M231 cells with targeted mutations in the *BRCA1* or *BRCA2* loci do not assemble significant numbers of RAD51 foci in response to 16-h treatment with 8 uM carboplatin. M231^*wt*^, M231 cells with intact HRR genes; M231^*BRCA1*^, cells with targeted mutations in *BRCA1*; M231^*BRCA2*^, cells with targeted mutations in *BRCA2* mean ± SEM calculated and significance determined by two-way ANOVA with Tukey’s adjustment for multiple testing. **B–E** Drug effects on final population sizes measured by SRB assay at day 6 of culture, means ± SD calculated and significance determined via 1-way ANOVA with Tukey’s adjustment for multiple testing. **F–I** Cell cycle profiles at 48 h of drug treatment as indicated. **B–I** Drugs: Deci,14 nM decitabine; Tala, 5 nM talazoparib; or 14 nM Deci + 5 nM Tala; or DMSO. **A–I** All experiments used at least triplicate samples, and were performed at least twice. ****, *p* < 0.0001; ***, *p* < 0.001; **, *p* < 0.01; ns, not significant

We tested for compromised HRR proficiency by assessing RAD51 repair foci increases in response to DNA damage by carboplatin treatment. Without treatment, foci numbers in the M231^*BRCA1*^ and M231^wt^ cells were similar (average 12.8, 10.7; *p* = 0.13), while the M231^*BRCA2*^ showed significantly more foci (versus M231^wt^ *p* = 1.3E−8; versus M231^*BRCA1*^; *p* = 2.2E−4; Fig. 1A). However while carboplatin treatment induced abundant new foci in M231^wt^ cells (*p* < 0.0001), foci numbers in M231^*BRCA1*^ and M231^*BRCA2*^ did not significantly increase. We conclude that we have generated HRR hypomorphic alleles in the M231 HRR-proficient background.

We tested the growth inhibitory effects of Tala with and without Deci in this cell line panel, using the triple negative breast cancer cell line SUM149PT as a control with a naturally occurring pathogenic *BRCA1* 2288delT mutation [33] in a different genetic background. We found that these drugs synergized to produce smaller final populations in all 4 cell lines in a 4 day endpoint SRB assay (M231^*wt*^, SynergyFinder ZIP score 23.39; M231^*BRCA1*^*,* ZIP score 16.37; M231^*BRCA2*^, ZIP score 12.82; SUM149PT, ZIP score 18.379; Supplementary Fig. S1C-F; [26]). These scores are well above the “likely synergistic” score boundary of > 10. As expected, at the SUM149PT IC_50_ doses, the *BRCA1* and *BRCA2*-targeted M231 cells were more sensitive to talazoparib than M231^*wt*^ cells (Fig. 1B–D) final population sizes: M231^*wt*^ Tala treated were 74% of controls versus M231^*BRCA1*^ ~ 48%, and M231^*BRCA2*^ 33%. They were also more sensitive to Tala + Deci treatment (M231^wt^ 29% of controls versus M231^*BRCA1*^ ~ 23%, and M231^*BRCA2*^**,** 16%). In SUM149PT controls this drug combination almost completely prevented growth (Fig. 1E; reduction to 4% of control treatment). Accordingly, prior studies testing whether this drug combination would be useful for HRR proficient tumors found synergistic M231^*wt*^ responses to Tala ± Deci, and Tala ± azacytidine in SRB endpoint assays [22].

Next, we found that BRCA mutations per se did not affect the ability of these mutants to transit the cell cycle normally under standard culture conditions (Fig. 1F-H; compare first columns, < 5% differences), and 48 h of either Tala or Deci produced only minor profile variations (Fig. 1F–H, second and third columns versus first). However in the drug combination, the M231^*wt*^ cells generated a larger S-Phase (from ~ 27 to ~ 40%,) and G2/M fraction (23% to 32%; Fig. 1F), suggesting that cells might be slowing DNA synthesis to repair drug-induced DNA damage when homologous recombination can be used to seamlessly mend DNA lesions. In contrast, M231^*BRCA1*^ and M231^*BRAC2*^ cells had increased the G2/M fraction (M231^*BRCA1*^ from 22 to ~ 40%; M231^*BRAC2*^ from 19 to 27%; Fig. 1G, H), similar to the expansion seen in SUM149PT (Fig. 1I; 21–47%)**.** G2/M stalling suggests that combination therapeutic treatment potentiates DNA damage, and that cells may be attempting DNA repair by inaccurate mechanisms available to cells with compromised HRR (review: [34]).

### Talazoparib plus decitabine treatment potentiates DNA damage in HRR deficient cells

**Fig. 2:**
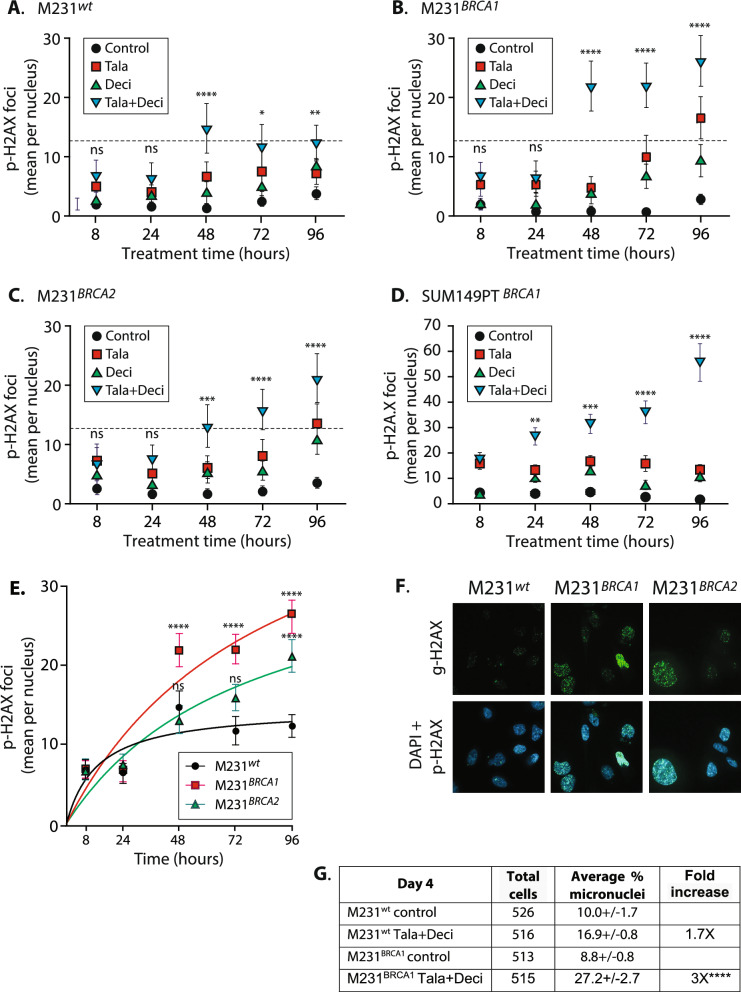
Talazoparib plus decitabine treatment potentiates cumulative DNA damage in HRR deficient cells. **A–D** p-H2A.X foci per nucleus in the M231 variants and in SUM149PT quantitated over 4 days. Each icon represents 50 nuclei analyzed in 3-D z-stack assemblies, dotted line, average foci numbers in Tala + Deci treated M231^wt^ cells from 48 h onwards. Means ± SEM shown, significant difference from Tala alone treatment determined by two-way ANOVA with Tukey’s adjustment for multiple testing. **E** Foci numbers from Tala + Deci treatment plotted to directly compare M231 BRCA variants. Curves fit using one site binding, nonlinear least squares fits**,** significant differences from M231^wt^ determined by two-way ANOVA, with Tukey’s adjustment for multiple testing, and indicated by asterisks over icons for M231^*BRCA1*^ and M231^*BRCA2*^. **A–E, G;** ****, *p* < 0.0001; ***, *p* < 0.001; **, *p* < 0.01; *, *p* < 0.1; ns, not significant. **F** Representative fluorescence images of p-H2A.X staining with talazoparib + decitabine treatment. **G** Percent of cells with micronuclei in control or Tala + Deci treatment visualized by DAPI staining of coverslips at treatment day 4. At least 500 cells per group examined. M231^wt^ versus M231^*BRCA1*^ foci increases significant at *p* = ****. **A–G** Drugs: Tala, 5 nM talazoparib; Deci, 14 nM decitabine; or Tala + Deci, 14 nM decitabine + 5 nM talazoparib; or Control, DMSO. All experiments except for **G** used at least triplicate samples, performed at least twice

We tested for increased DNA damage signaling by counting phosphorylated histone H2A.X (p-H2A.X) foci in our isogenic M231 cell lines and in SUM149PT control cells over a 4 day time course. As expected from their ability to accurately repair DNA, the HRR proficient M231^*wt*^ cells maintained a relatively low, tonic level of p-H2A.X foci at all timepoints and tratments, only briefly rising over 10 per cell at 48 h with Tala + Deci (Fig. 2A, blue, ~ 12 per nucleus average). In contrast, p-H2A.X foci numbers in the M231^*BRCA1*^, M231^*BRCA2*^ and the naturally occurring mutant SUM149PT treated with Tala + Deci increased significantly over Tala alone from 48 h onwards (Fig. 2B–D, blue versus red icons; asterisks: significant difference between Tala + Deci versus Tala).

Comparison of Tala + Deci responses between the M231 isotypes revealed significantly more p-H2A.X foci in M231^*BRCA1*^ cells from 48 h onwards, and in the M231^*BRCA2*^ cells at 96 h (Fig. 2E, F example images, complete image panel Supplementary Fig. S2). This signaling was accompanied by an increase in cells with abnormal chromatin morphology and content, including a 3.5 fold increase in cells with micronuclei in M231^*BRCA1*^ versus a 1.5 fold increase in the M231^*wt*^ cells at treatment day 4 (Fig. 2G).

### Talazoparib plus decitabine treatment potentiates death in BRCA mutant cells

**Fig. 3:**
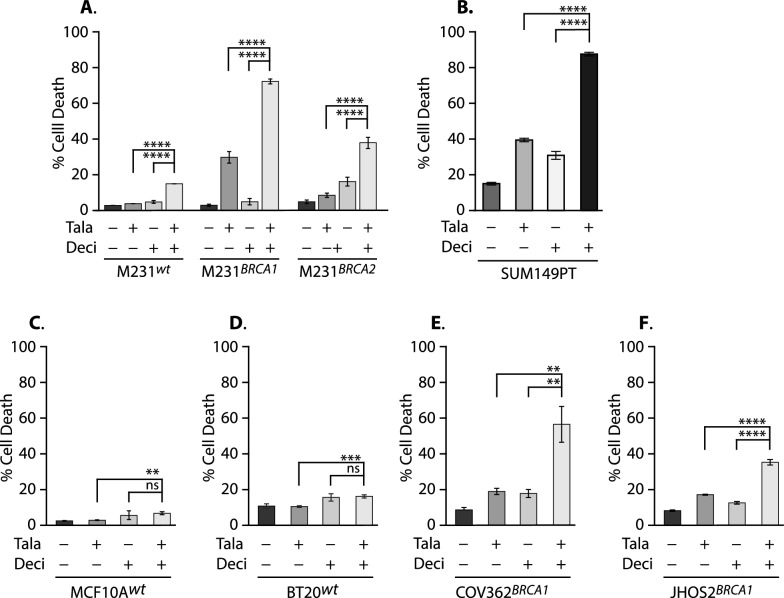
Talazoparib plus decitabine treatment potentiates death in BRCA mutant cells. **A–F** Death analysis using Calcein AM versus Propidium Iodide (PI) staining at day 6 of drug treatment. Values indicate mean ± SD of PI positive cells. **A, B** Cell death in the M231 isogenic variants, and in SUM149PT. **A** Significance determined by two-way ANOVA with Tukey’s adjustment for multiple testing. **C, D** Little cell death in the HRR proficient non-transformed TNBC line MCF10A or the HRR proficient breast cancer cell line BT20. **E, F** Death in BRCA1 mutant ovarian cancer cell lines COV362; JHOS2. **B–F** significance determined by one-way ANOVA with Tukey’s adjustment for multiple testing. **A–F** ****, *p* < 0.0001; ***, *p* < 0.001; **, *p* < 0.01; ns, not significant. All experiments use at least triplicate samples, and performed at least twice. Drugs: Tala, 5 nM talazoparib; Deci, 14 nM decitabine

Endpoint assays such as Sulforhodamine B or 3-(4,5-dimethylthiazol-2-yl)-5-(3-carboxymethoxyphenyl)-2-(4-sulfophenyl)-2H-tetrazolium (MTS) cannot determine whether cell population size differences at the end of an assay are due to cell cycle arrest/slowing or cell death. To make this distinction, we directly tested the effects of BRCA mutation on drug-induced cell death in our M231 variants using the Calcein AM Live and Dead Cell Assay in which live cells generate a green-fluorescent dye, while dead cells stain with the nucleic acid intercalating dye propidium iodide. We found that Tala + Deci treatment produced significantly more death than treatment with either drug alone regardless of *BRCA* allele status (Fig. 3A; each isotype *p* < 0.0001). But in M231^wt^ cells, the combination effects were minimal (16% maximum death), while death in the *BRCA1* mutants was 30.7% with Tala and increased to 73% with Tala + Deci. In *BRCA2*-targeted cells, Tala-induced death was 9.6% and increased to 39% in combination with Deci. Similarly, in SUM149PT Tala- induced death was 41%; Deci 32.6%; and the drug combination killed 89% of cells (Fig. 3B. *p* < 0.0001 each comparison; Supplementary Fig. S3 representative FACS plots). Given that the M231 variants were isogenic, these data directly implicate BRCA1 and BRCA2 mutation in the death response.

This was further supported by the observation of minimal death in an HRR proficient non-transformed breast cell line (Fig. 3C; MCF10A; > 95% live with any treatment), or in another HRR proficient breast cancer cell line (Fig. 3D; BT20, 17% maximum death). In contrast, Tala + Deci also potentiated death in two independent BRCA1 mutant ovarian cancer cell lines (Fig. 3E**;** COV362, up to ~ 58% death**;** and Fig. 3F; JHOS2 up to 35% death). Thus, the enhanced death phenotype we saw in our isogenic M231-derived variants was not limited to the M231 genetic background, or to breast cancer. We note that SUM149PT was always more sensitive to all drug treatments, suggesting that it may also express other modifiers that further potentiate these responses.

In subsequent studies we observed minimal and insignificant increases in Annexin V + /PI- staining at various timepoints in response to Tala + Deci in all M231 variants (data not shown).

### Decitabine co-treatment allows the use of lower talazoparib doses to affect similar therapeutic outcomes

**Fig. 4:**
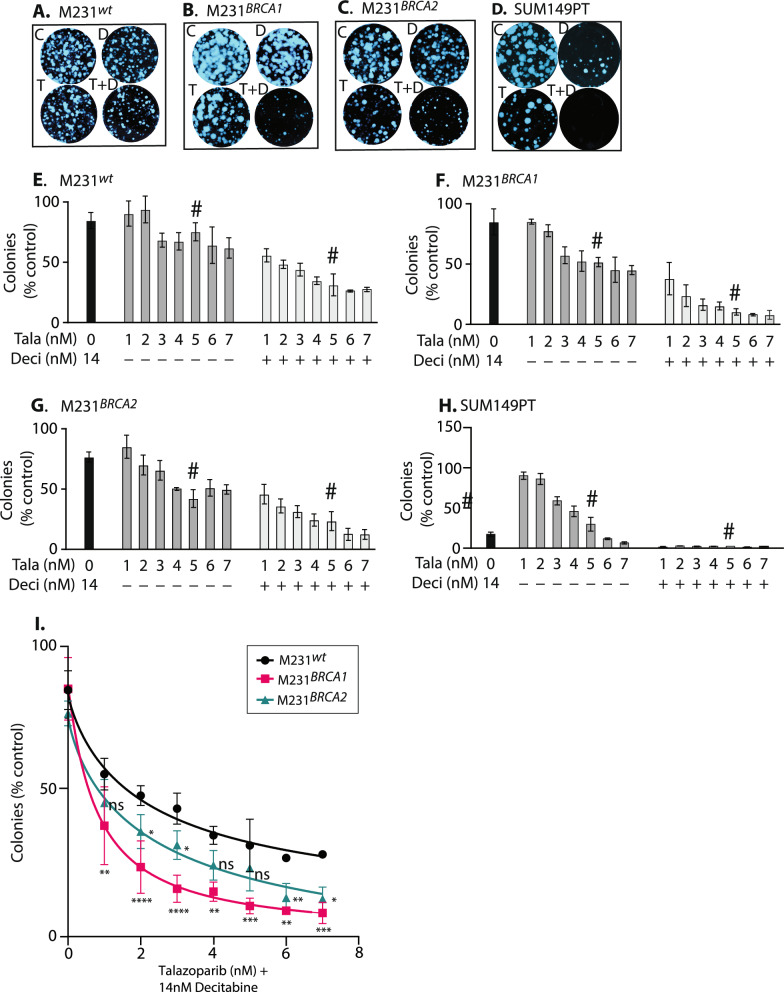
Colony formation attenuated by treatment with talazoparib + decitabine. **A–D** Representative crystal violet stained culture plate images at day 14, illustrating the effects of Tala, Deci, and combination treatment on colony growth for each M231 *BRCA* isotype and SUM149PT. Drugs: C, DMSO; T, 5 nM talazoparib; D, 14 nM decitabine; T + D, both drugs. **E–H** Deci treatment reduces the colonies formed at each Tala dose in all M231 isotypes and SUM149PT. (# indicates the 5 nM Tala ± Deci values for each cell line for comparison). Colony quantitation used BioRad ChemiDoc imaging System (BioRad®), analyzed by ImageJ, values indicate mean ± SEM**. I** Values from Tala + Deci treatment plotted to directly compare M231 BRCA variants. Non-linear curve fit, asterisks below red icons represent significant differences for M231^*BRCA1*^ versus M231^*wt*^ cells, and asterisks beside green icons represent significant differences for M231^*BRCA2*^ versus M231^*wt*^ cells. Significance determined by two-way ANOVA with Tukey’s adjustment for multiple testing. ****, *p* < 0.0001; ***, *p* < 0.001; **, *p* < 0.01; *, *p* < 0.1; ns, not significant. All experiments used at least triplicate samples, and performed at least twice. Prior studies testing colony formation effects of 150 mM azacytidine + 10 nM Tala in M231^*wt*^ cells concur with our results. Combination treatment reduced colonies to ~ 60% of controls, while in their SUM149PT controls, colonies were reduced to about 30% of controls

Clinical studies suggest that PARPi have toxicities often requiring dose reduction to ineffective concentrations. We tested whether the addition of a DNMTi could allow use of lower dose PARPi, using colony formation assays. We found that treatment with our standard drug dose combination (5 nM Tala, 14 nM Deci) reduced colony formation for all M231 variants and the independent BRCA1 mutant cell line SUM149PT, but with more limited effects on MDA-231^wt^ cells (Fig. 4A–D representative images; E–H, # icon is over standard Tala (5 nM) doses with and without 14 nM Deci**)**. This is concordant with the cell number reductions seen in SRB assays, and with the death response data in Fig. 3.

In Tala titration studies, Deci addition reduced colony formation at each Tala dose in all M231 variants (Fig. 4E–G), and potently in SUM149PT -where Tala doses could be reduced to 1 nM (IC_50_ 5.2 nM as single agent), and co-treatment with Deci still strongly inhibited colonies (Fig. 4H). Direct comparisons between M231 isotypes revealed the MDA-231^*BRCA1*^ cells were significantly more sensitivity to Deci + low dose Tala than M231^wt^ cells (Fig. 4I**,** from 1 to 7 nM**;** red icons and curve fit, asterisks indicate *p*-values from 0.001 to 0.0001). All effects were more pronounced in M231^*BRCA1*^ cells than in M231^*BRCA2*^ mutants. We conclude tht Tala + Deci treatment impairs the ability of cells to produce viable colonies, and that this drug sensitivity is enhanced by BRCA mutation. We note that others reported cooperative colony reduction in MDA-231^wt^ cells treated with azacytadine doses ranging from 100 to 400 nM combined with Tala doses ranging from 5 to 20 nM [22].

### *Talazoparib* + *decitabine treatment significantly inhibits xenograft and PDX growth*

**Fig. 5:**
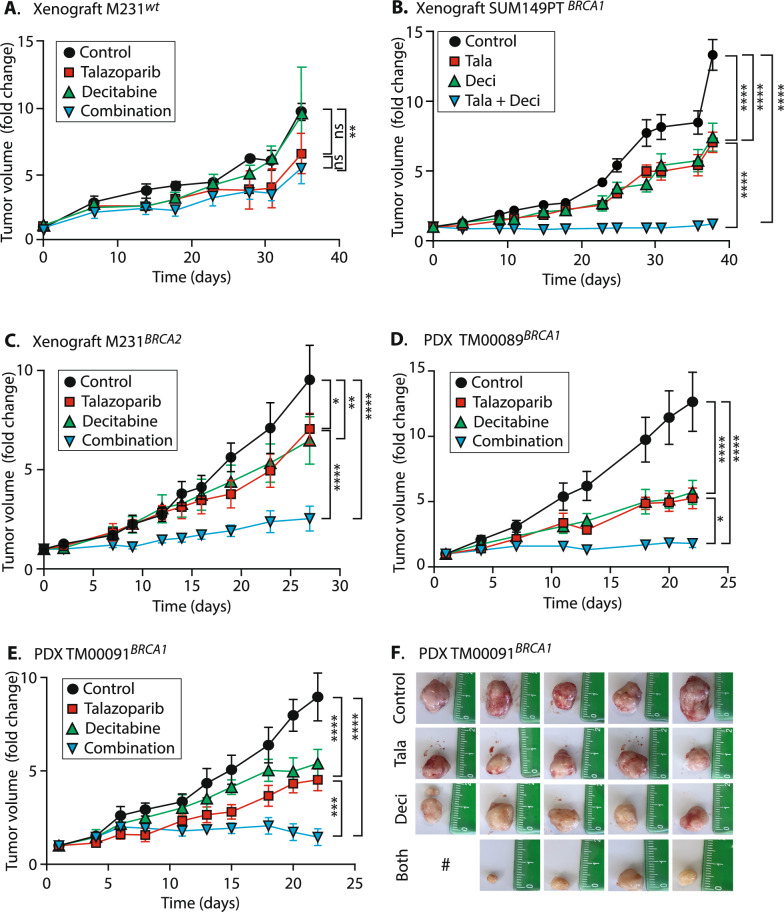
Talazoparib plus decitabine treatment significantly impair growth of BRCA mutant xenografts and PDX. **A** M231^*wt*^ xenografts. **B** SUM149PT xenografts. **C** M231^*BRCA2*^ xenografts. **D** PDX TM00089, n = 6 animals per group. **E** PDX TM00091. **A, B, C, E** n = 5 animals per group. **A–F** Xenografts recieved 0.1 mg/kg talazoparib (Cat. #S7048, SelleckChem®) given by oral gavage or carrier, and/or 0.2 mg/kg decitabine (Cat. #S1200, SelleckChem®) or carrier given by intraperitoneal (IP) injection. PDX received the identical talazoparib dose, and ½ dose of decitabine. Treatment schedule for all mice was 5-days on/2-days off. **A–E** Values indicate mean ± SEM**.** Significance determined by two-way ANOVA with Tukey’s adjustment for multiple testing. ****, *p* < 0.0001; ***, *p* < 0.001; **, *p* < 0.01; *, *p* < 0.1; ns, not significant. **E** final tumor images for TM00091; #, animal died 2 days before tumor harvests, tumor was unrecoverable

We tested the effects of clinically relevant concentrations of talazoparib and decitabine [35] in xenografts and PDX models. At these doses, the M231^*wt*^ xenografts showed no effects of Deci treatment (Fig. 5A green versus black lines) and Deci did not add benefit to Tala treatment (red versus blue lines). In contrast, in SUM149PT xenografts, each drug reduced tumor volumes ~ 50% relative to controls (Fig. 5B; red, green, versus black lines**)** and combined, they produced tumors that were only ~ 9% of controls** (**blue; *p* < 0.0001, all comparisons). Xenografts of these two cell lines had been previously shown to be moderately sensitive to Azacytidine + Tala [22], but with much smaller growth inhibition, which we speculate may have to do with the use of azacytidine instead of decitabine.

In M231^*BRCA2*^ variant xenografts, Deci and Tala significantly reduced growth versus control treatment (Fig. 5C; Tala, red, *p* = 0.03; Deci, green, *p* = 0.002), and their combined efficacy was significantly greater than that of either individual drug (blue, *p* < 0.0001 versus all others). Efficacy was also tested in two independent BRCA1 mutated breast cancer PDX models. PDX TM00089 is derived from a patient with a germline pathogenic v757fs*BRCA1 mutation (truncation) who had received prior cisplatin/taxol therapy for a fallopian tube carcinoma ([31]; Mouse Models of Human Cancer Database, Jackson Research Labs). TM00089 growth was sharply reduced by either drug treatment (Fig. 5D; red, green, *p* < 0.0001 for each) and was nearly halted by Tala + Deci (blue, *p* < 0.0001).

The second BRCA1 PDX, TM00091, expresses the common pathogenic C61G missense mutation, with 100% variant allele frequency. This decreases BRCA1-BARD1 heterodimer formation and E3 ubiquitin ligase activity [36], with minimal response to cisplatin [37]. We found that TM00091 PDX were also extremely sensitive to Tala + Deci, exhibiting little tumor growth over the treatment course** (**Fig. 5E, blue *p* < 0.0001 versus controls; F, dissected tumors**)** which is significantly improved control over treatment with either drug alone (versus talazoparib, red, *p* = 0.0003; versus decitabine, green, *p* < 0.0001). In all xenograft and PDX experiments, the drug combination was well tolerated, as demonstrated by animal behavioral observations and weights (Supplementary Fig. S4A–E).

## Discussion

Enthusiasm for combined DNMTi + PARPi therapeutics arose from observations that both DNMT inhibitors and PARP inhibitors trap their respective targets on DNA at sites of exogenously induced damage, preventing or reducing repair [22]. Preclinical studies using treatment with camptothecin, methyl methanesulfonate, irradiation, or laser-induced DNA damage demonstrated Tala and Deci interaction in solution and cooperative, enhanced DNA binding at sites of DNMTi incorporation [22, 24]. PARP1-DNMT1 foci were found to be larger and bound DNA more tightly than PARPi-PARP1 complexes, in principle providing a more significant DNA repair disruption [22].

In efforts to bring such novel combinations to patients with HRR proficient tumors and expand the utility of PARPi therapy, various PARPi + DNMTi combinations have been tested in preclinical models of HRR proficient TNBC, AML, non-small cell lung cancer, and ovarian cancer [22–24]. Improved growth inhibition by the combination over PARPi treatment alone in several preclinical models prompted phase 1 clinical trials in leukemia [25] and in breast cancer (ClinicalTrials.gov Identifier: NCT04134884). The combination was well tolerated at approved doses with either drug in the AML study, perhaps not surprising as myelosuppression is typically not evaluable in AML. However the study showed minimal anti-tumor efficacy [25], which would be predicted from our studies in tumor models with intact HRR status. These patients were not selected for HR mutations. The second trial with this combination in TNBC patients with intact HR status is near completion, and also shows limited actual benefit (personal communication). This trial was different in its goals to keep the concentration of the oral DNMT inhibitor (decitabine/cedazuridine combination; ASTX727) high. Our data provides novel insights in the effects of this drug combination to allow better selection of patients who may benefit.

We hypothesized that inhibition of PARP plus inhibition of DNMT1 would most likely benefit patients with HRR deficient tumors, since these tumors repeatedly undergo erroneous DNA repair during treatment exposure. Our studies support this premise. Our finding of more baseline RAD51 foci in the BRCA2 mutants than the wildtype or BRCA1 mutants is reminiscent of other observations that *bona fide* BRCA2 mutants can assemble at least some RAD51 foci [38]. Our BRCA2 hypomorph may retain such weak potential, however it is clearly unable to form significant numbers of new foci upon DNA damage by the therapeutic carboplatin.

Similar to previous reports, we find that the combination of Tala and Deci in HRR proficient cells induces some anti-proliferative effects (Figs. 1B, 4A, E, I, 5A). However, we found little to no actual cell death in HRR proficient cells (Fig. 3A, C, D), suggesting that this drug combination may provide limited benefit for patients with intact HRR capacity, or may require concentrations that exceed clinically approved concentrations of each drug. This may at least in part be due to the narrow therapeutic window of PARPi drugs. Several iterations of increasing the PARP or the DNMT inhibitors did not overcome the lack of apoptosis in the wild type cells (data not shown).

In contrast, M231 isogenic, genetically mutated cells (Fig. 3A), and intrinsically mutated cells with HRR deficiency (Fig. 3B, E, F) exhibited significantly more cell death in vitro, yielding improved tumor control in colony forming assays (Fig. 4I). Similarly, pronounced anti-tumor effects were seen in xenografts and PDX models with BRCA mutations (Fig. 5B–F) with little impact on animal well-being and weight. Titrations of Tala in our colony formation assays (Fig. 4E–I) suggests that the Tala + Deci combination could be given at low PARPi concentrations, potentially reducing clinically necessary doses and thus limiting unwanted side effects.

Unlike other studies, we measured DNA damage from low dose Tala + Deci treatment without using a third, DNA damaging agent. We found that DNA damage accumulates in BRCA mutated cells over time (Fig. 2B–G), thus we are most likely impacting PARPi + DNMTi complex activity at replication forks, where DNMT and PARP are in natural proximity and functionally required [10, 39]. From our results and previous work by others [22], we propose that this drug combination produces complexes that increase the frequency of fork collapse into DSBs, increasing p-H2A.X foci numbers and DNA damage. Importantly, inaccurately repaired DNA in BRCA-compromised cells will either trigger death during mitosis, or induce further damage during subsequent replication cycles, until fatal amounts of DNA damage are generated. In contrast, HRR intact cells have the ability to accurately repair damage during each S-Phase to maintain low damage levels and thus preserve viability. In support of this model, early studies of DNMTi treatment effects reported induction of DNA damage signaling, apoptosis, and production of aberrant DNA structures that are most constant with replication fork collapse and which require HRR for correct resolution [40]. Similarly, seminal studies of PARP function found that during normal replication, essentially all PARP1 and PARP2 activities occur at replication forks to bind and mend un-ligated Okazaki fragments [10]. This places the coordinated activity of PARP1, DNMT1, and their inhibitors at replication forks.

Other activities of DNMTi that can cooperate with PARP inhibition include the re-activation of tumor suppressors [41]; reduction of MutL-alpha and MutS alpha DNA mismatch repair protein levels [42]; and reduction of low fidelity DNA repair pathways such as non-homologous end joining (NHEJ), alternative non-homologous end joining (alt-NHEJ), and intra-strand crosslink repair [24]. While these may also influence the therapeutic responses that we see, this would occur in all M231 variants including the M231^*wt*^ parental cells. Thus, the stark contrast in cell death between the parental M231^*wt*^ cells and the isogenic *BRCA*-targeted variants strongly argues for the dominant importance of BRCA1 and BRCA2 mutations in cell death. We believe that this is the first report of potentially clinically critical differences in the levels of death induced in HRR deficient, versus HRR proficient tumor cells in response to this drug combination, and speculate that clinically, the drug combination will preferentially benefit BRCA mutant cancers to enhance responsiveness to PARPi.

Currently we cannot gauge the variety or prevelence of BRCA mutant tumors that will respond so completely to treatment with the Tala + Deci combination. However, in a 2009 genetic analysis of *BRCA1* and *BRCA2* sequences from 46,276 women, the C61G mutation was found to be prevelant in 2.3% of western europeans, and 6.4% of central europeans [43]. C61G is one of three most common founder mutations in the polish population [44]. Thus our PDX experimental result with TM00091, which bears the C61G mutation, may not be entirely irrelevant. Other significant genetic variants in TM00091 and TM00089 are included in Supplemental Table 1, and it is possible that they may ultimately contribute to the definition of a more exact susceptibility signature.

Whether these findings will translate to other HR mutation including PALB2, ATM and CHEK2 is being evaluated in a clinical trial allowing the enrollment of  BRCA1 or 2, ATM, PALB2 and CHEK2 in a combination of a PARP and DNMT inhibitor (Munster, PI UCSF).

## Conclusions

We conclude that low dose DNA methyltransferase inhibition can cooperate with low dose PARP inhibition to potentiate DNA damage and cell death specifically in HRR deficient cells. This combination also ultimately produces better tumor control than treatment with PARP inhibitors alone. We predict that clinical benefit of such a drug combination will more likely be apparent in patients with DNA repair defective tumors, and suggest to focus clinical exploration of this drug combination in these patients, with the goals of enhancing tumor cell death at minimal toxicities and avoidance of long term sequelae.

## Supplementary Information

Additional file1 (PDF 4648 KB)

Additional file2 (PDF 5425 KB)

Additional file3 (PDF 1972 KB)

Additional file4 (PDF 1964 KB)

Additional file5 (XLSX 11 KB)

## Data Availability

No datasets were generated or analysed during the current study.

## Abbreviations

aa: Amino acid
AML: Acute myelogenous leukemia
BRCA1: BReast CAncer gene 1
BRCA2: BReast CAncer gene 2
CpG: Cytosine nucleotides immediately 5′ of guanines
Deci: Decitabine
DAPI: 4′,6-Diamidino-2-phenylindole
DNMT: DNA methyltransferase
DNMTi: DNMT inhibitor
DSB: DNA double strand break
HRR: Homologous recombination repair
M231: Breast cancer cell line MDA-MB-231
M231^*wt*^: Breast cancer cell line MDA-MB-231 with intact HRR capacity
M231^*BRCA1*^: Breast cancer cell line MDA-MB-231 with targeted mutation in the BRCA1 gene
M231^*BRCA2*^: Breast cancer cell line MDA-MB-231 with targeted mutation in the BRCA1 gene
PARP: Poly (ADP-Ribose) polymerase
PARPi: PARP inhibitor
p-H2A.X: Phosphorylated-histone H2A.X
PDX: Patient Derived Xenograft
SRB: Sulphorhodamine B endpoint assay
Tala: Talazoparib
TNBC: Triple negative breast cancer
