# Supplementary material for: Low dose DNA methyltransferase inhibitors potentiate PARP inhibitors in homologous recombination repair deficient tumors
Source: Breast Cancer Res. 2025 Jan 16;27:8. doi: 10.1186/s13058-024-01954-y (PMC11740508; doi:10.1186/s13058-024-01954-y)
Supplement: Supplementary file 1 — Additional file1 (PDF 4648 KB) [file 13058_2024_1954_MOESM1_ESM.pdf]

S1A.

Anchor Sequence: CCTTGGAACTGTGAGAACTC UCRP231B1.85

Degenerate Sequence: [AT] [CG] A [TG] [TG] [AG] [AC] A [TA] [TA] G [CG] G [TG] A

Allele 1: CCTTGGAACTGTGAAGAACTCACATTGAATTGGGTA

Allele 2: CCTTGGAACTGTGAGAACTCTGAGGACAAAGCGGA

Alignments of Allele1, Allele 2, and reference sequence:

Allele 1: CCTTGGAACTGTGAGAACTC ------------------------------(42-bp deletion)--------------------------ACATTGAATTGGGTA

Allele 2: CCTTGGAACTGTGAGAACTCTGAGGACAA-(3)--AGCGGA

Reference: CCTTGGAACTGTGAGAACTCTGAGGACAAAGCAGCGGATACAACCTCAAAAGACGTCTGTCTACATTGAA

S1B.

Anchor Sequence: GTCTAATTTGAATTTATCTACAGCGGCAGAGTCAGCCCTT UCRP231B2A

Degenerate Sequence: [AC] [TA] [TG] C [TG} [CG] C [TA] [TG] [GA] [CG] TC [TA] [TG] [TC] [GC] [AC] [AT] T

Allele 1: GTCTAATTTGAATTTATCTACAGCGGCAGAGTCAGCCCTT

Allele 2: GTCTAATTTGAATTTATCTAATTCTCCTTGCTCTTTGAAT

Alignments of Allele 1, Allele 2, and reference sequence:

Allele 1: GTCTAATTTGAATTTATCTA-----(22-bp deletion)-------------CAGCGGCAGAGTCAGCCCTT

Allele 2: GTCTAATTTGAATTTATCTAATTCT----------------(33-bp deletion)-------------------------CCTTGCTCTTTGAAT

Reference: GTCTAATTTGAATTTATCTAATTCTTTTACAGGAGATTGGTACAGCGGCAGAGTCAGCCCTTGCTCTTTG

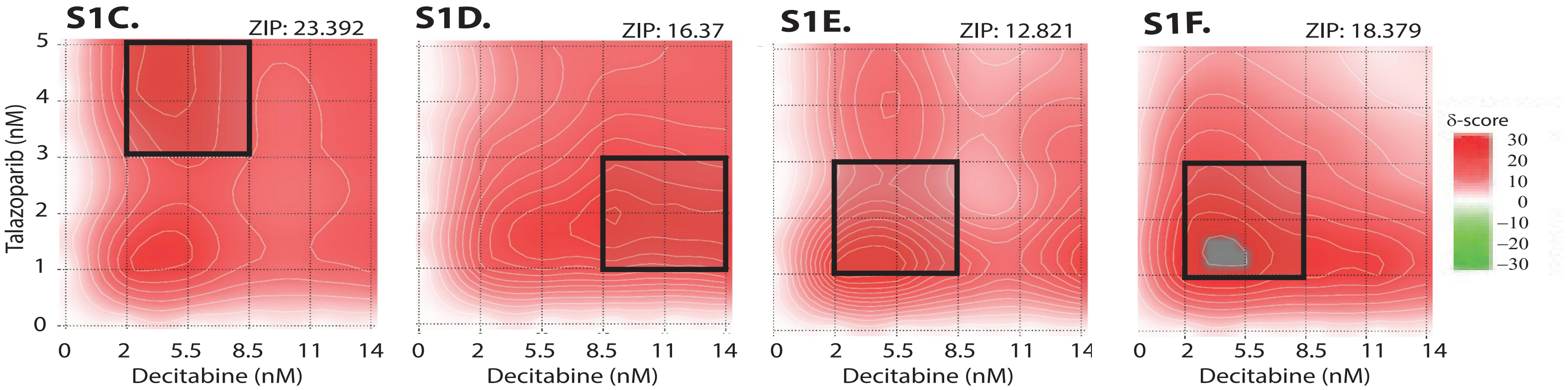

Supplementary Fig. S1. Analysis of CRISPR mediated deletions in the BRCA1 and BRCA2 genes in the triple negative breast cancer cell line MDA-MB-231 (M231). A. DSDecodeM analysis of the BRCA1 gene sequenced from clone 1.85, revealing a 42bp deletion and a 3bp deletion in each BRCA1 allele respectively. B. DSDecodeM analysis of the BRCA2 gene sequenced from clone 54, revealing a 22bp and a33 bp deletion in each BRCA2 allele respectively. C-E. Drug interaction heatmaps for combinatorial talazoparib (0-5nM) and decitabine (0-14 nM) treatment ; C. M231 wt cells ($M231^{wt}$); D. M231 BRCA1-targeted clone1.85 ($M231^{BRCA1}$); E. M231 BRCA2-targeted clone 54 ($M231^{BRCA2}$); F. SUM149PT with endogenous BRCA1 mutation.
