# Supplementary material for: Low dose DNA methyltransferase inhibitors potentiate PARP inhibitors in homologous recombination repair deficient tumors
Source: Breast Cancer Res. 2025 Jan 16;27:8. doi: 10.1186/s13058-024-01954-y (PMC11740508; doi:10.1186/s13058-024-01954-y)
Supplement: Supplementary file 2 — Additional file2 (PDF 5425 KB) [file 13058_2024_1954_MOESM2_ESM.pdf]

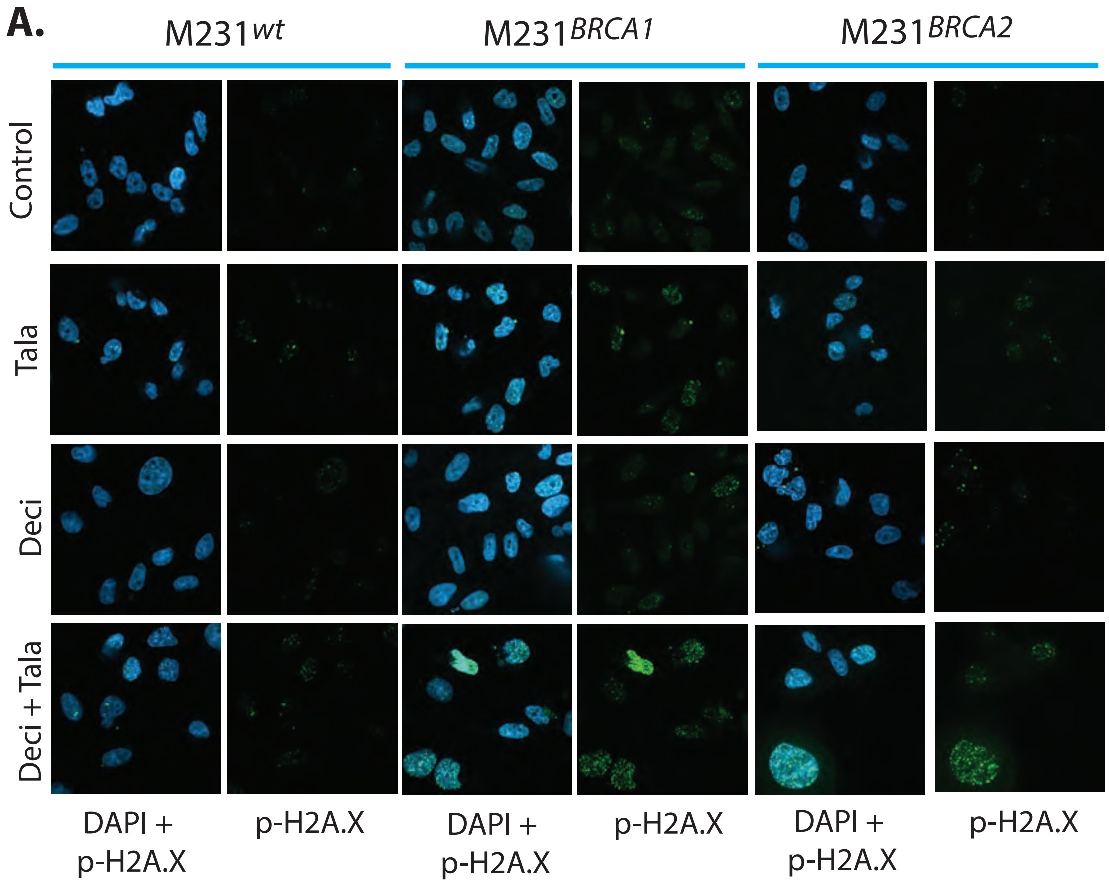

Supplementary Fig. S2. Representative immune fluorescence images of phosphorylated H2A.X. p-H2A.X foci (green) in nuclei (blue) of cells in each treatment group at 96 hours.
