# Supplementary material for: Low dose DNA methyltransferase inhibitors potentiate PARP inhibitors in homologous recombination repair deficient tumors
Source: Breast Cancer Res. 2025 Jan 16;27:8. doi: 10.1186/s13058-024-01954-y (PMC11740508; doi:10.1186/s13058-024-01954-y)
Supplement: Supplementary file 3 — Additional file3 (PDF 1972 KB) [file 13058_2024_1954_MOESM3_ESM.pdf]

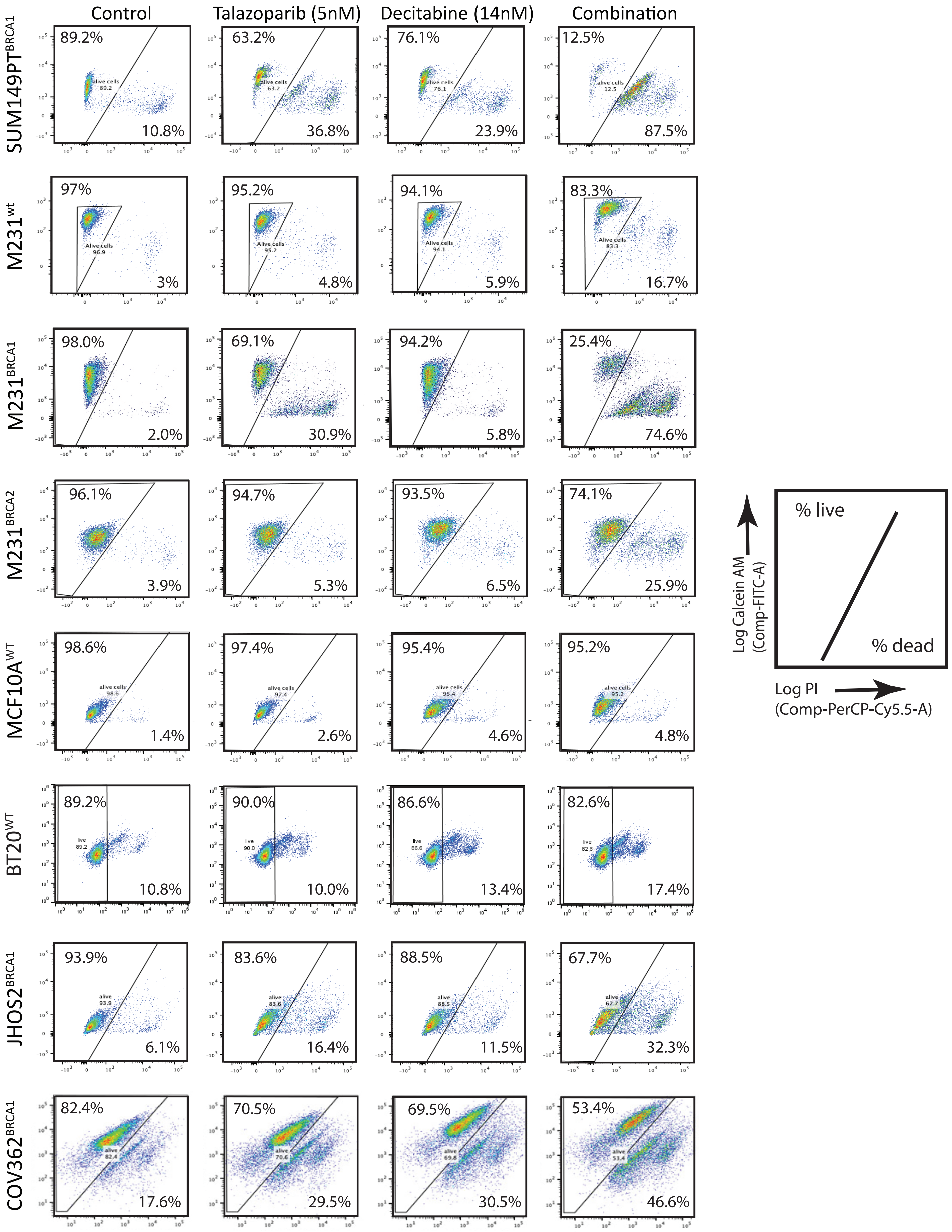

Supplementary Fig. S3. Representative FACS plots of cells treated with the indicated therapeutics for 6 days, stained for live versus dead cells. Vertical axis: live cell label, Calcein AM (fluorescence at 530/30); horizontal axis dead cell label: Propidium Iodide (PI; 670 LP). Each condition n=3, experiments performed at least twice. Representative experiments shown.
