# Supplementary material for: Low dose DNA methyltransferase inhibitors potentiate PARP inhibitors in homologous recombination repair deficient tumors
Source: Breast Cancer Res. 2025 Jan 16;27:8. doi: 10.1186/s13058-024-01954-y (PMC11740508; doi:10.1186/s13058-024-01954-y)
Supplement: Supplementary file 4 — Additional file4 (PDF 1964 KB) [file 13058_2024_1954_MOESM4_ESM.pdf]

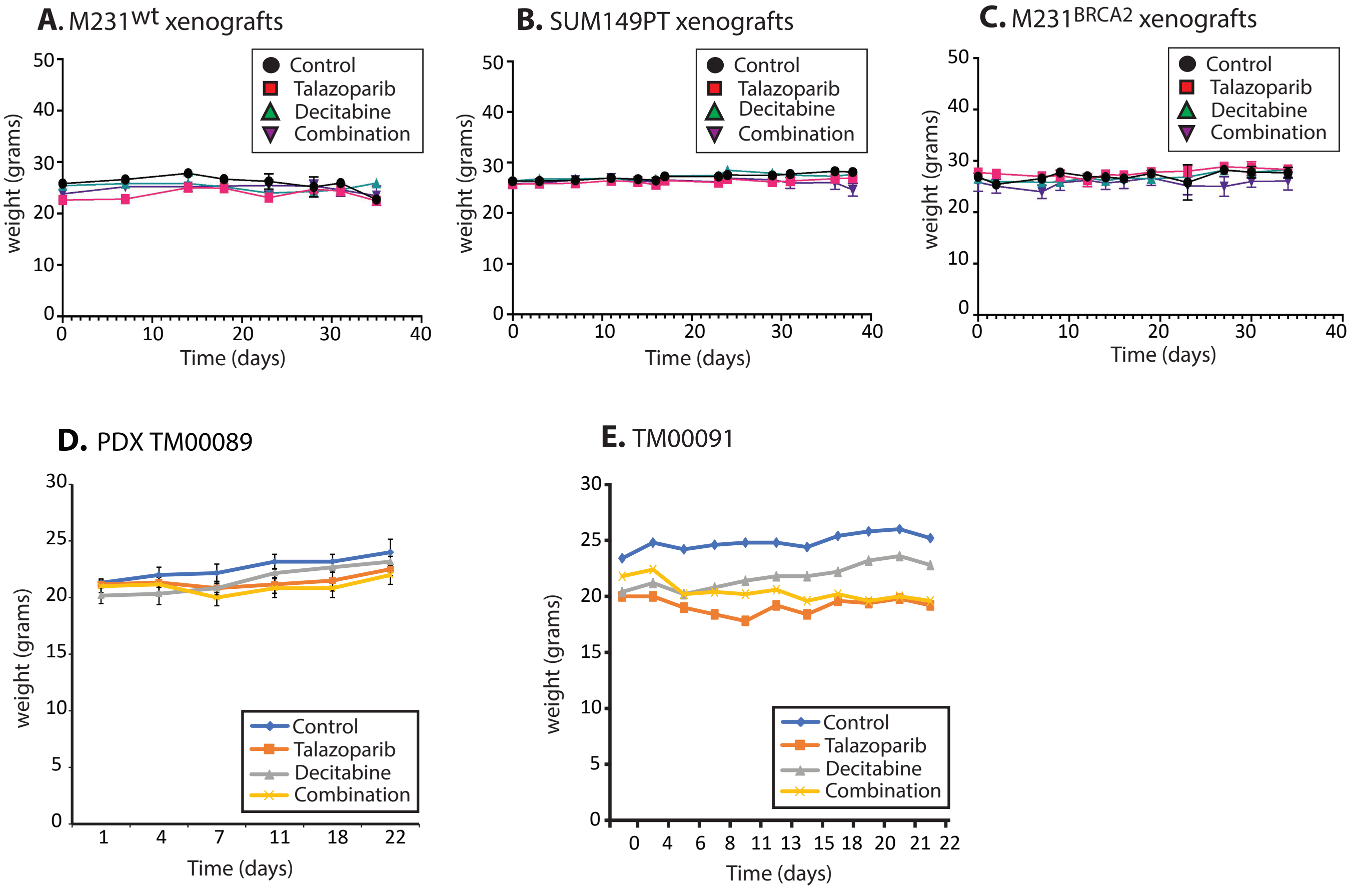

Supplementary Fig. S4. Mice in the xenograft and PDX experiments of Fig. 5 A-E maintain normal weights throughout the experimental timecourse.
